# Traditional Chinese medicine based on Tongjiang methodology combined with proton pump inhibitor (PPI) step-down in treating non-erosive reflux disease: a study protocol for a multicentered, randomized controlled clinical trial

**DOI:** 10.1186/s13063-022-06811-x

**Published:** 2022-10-18

**Authors:** Xia Li, Haomeng Wu, Beihua Zhang, Ting Chen, Xiaoshuang Shi, Jinxin Ma, Jiaqi Zhang, Xudong Tang, Fengyun Wang

**Affiliations:** 1 Department of Spleen and Stomach Diseases of Traditional Chinese Medicine, China-Japan Friendship Hospital, Beijing, 100029 China; 2 The Second Clinical College of Guangzhou University of Chinese Medicine, Guangzhou, 510120 China; 3 Department of Gastroenterology, Xiyuan Hospital Affiliated to China Academy of Traditional Chinese Sciences, Beijing Institute of Spleen and Stomach Disease of Traditional Chinese Medicine, Beijing, 100091 China; 4 Department of Gastroenterology, The Third Affiliated Hospital of Beijing University of Chinese Medicine, Beijing, 100020 China; 5 Department of Gastroenterology, Peking University Traditional Chinese Medicine Clinical Medical School (Xiyuan), Beijing, 100091 China; 6 China Academy of Traditional Chinese Sciences, Beijing Institute of Spleen and Stomach Disease of Traditional Chinese Medicine, Beijing, 100091 China

**Keywords:** Nonerosive reflux disease (NERD), TCM granules, PPI, Randomized controlled trial (RCT), Protocol

## Abstract

**Background:**

Non-erosive reflux disease (NERD) is characterized by typical gastroesophageal reflux symptoms, such as heartburn and regurgitation but an absence of esophageal mucosal damage during upper gastrointestinal endoscopy. Although proton pump inhibitors (PPIs) are the first line therapy, almost 50% of patients with NERD fail to respond to this treatment. Traditional Chinese medicine (TCM) can better relieve the symptoms of NERD. Therefore, a randomized controlled trial (RCT) was designed to investigate the efficiency of TCM granules based on Tongjiang (TJ) methodology combined with PPI step-down therapy for NERD patients who did not respond to PPIs alone.

**Method:**

This multicentered, double-blinded, RCT with two parallel groups will recruit 174 participants who will be randomized into the TCM granules combined with PPI step-down group (*n* = 87) and the TCM granules placebo combined with PPI step-down group (*n* = 87). Both groups of participants will receive 6 weeks of treatment and 4 weeks of follow-up, and all participants will be assessed for related symptoms, mental health status, and quality of life at each visit. The primary outcome measurements include visual analog scale (VAS) for heartburn and regurgitation and the major symptoms scale. The secondary outcome measurements include PPI withdrawal rate, symptom recurrence rate, minor symptoms scale, SF-36, PRO, SAS, SDS, GERD–HRQL, and TCM syndromes scales.

**Discussion:**

Previous research has shown that TCM is capable to alleviate NERD symptoms. This trial will help to provide a better understanding of the synergistic efficiency of the combination of TCM and PPIs, to explore whether the dosage of PPIs can be reduced after the supplement of TCM granules and to provide a feasible plan to reduce dependencies or withdraw NERD patients from PPIs. The outcome of this trial is expected to reduce the symptom recurrence rates, lessen patients’ physical and psychological burdens, and achieve good social benefits.

**Trial registration:**

Clinicaltrials.gov NCT04340297. Registered on April 9, 2020

## Background

Gastroesophageal reflux disease (GERD) is one of the most common chronic progressive upper gastrointestinal tract disorders of the esophagus, characterized by heartburn and regurgitation symptoms [1]. The prevalence of this disease ranges from 2.5% to more than 25% according to individual cross-sectional surveys [2]. GERD can be divided into several phenotypes: erosive esophagitis (EE), non-erosive reflux disease (NERD), and Barrett’s esophagus (BE) [3, 4]. NERD is the most frequent phenotype of gastroesophageal reflux disease (GERD). Rome IV consensus defines NERD as “the presence of abnormal acid exposure time (AET) with or without reflux–symptom association on ambulatory reflux monitoring performed off anti-secretory therapy” [5]. It accounts for approximately 70% of GERD patients and is characterized by typical symptoms and the lack of any visible endoscopic findings of esophageal mucosal lesions [6, 7]. The majority of patients with NERD have weakly acidic reflux [8]. Some studies have shown that the function of the esophageal mucosal barrier is not significantly altered in NERD patients [9]. NERD is a refractory gastrointestinal disease, and patients often need long-term treatment to control their symptoms. Proton pump inhibitors (PPIs), a type of acid-suppressive drug, are considered the first-line treatment drugs for NERD. There are obvious deficiencies in treatment guideline and safety of PPIs, which demonstrated the following problems in clinical practice: approximately 50% of patients with weakly acidic reflux respond partially or not at all to PPIs [10]; 20–30% of patients treated with a standard PPI dose for troublesome GERD symptoms experience persistent heartburn and/or regurgitation [11, 12], and more than 75% NERD patients may experience symptomatic relapse within 6 months of stopping treatment so they receive long-term treatment to control symptoms [13, 14]; long-term use of PPIs can cause many adverse events, such as indigestion, fundic gland polyps, atrophic gastritis, and gut microbiota imbalance [15, 16], as well as PPI resistance or dependence. This life-long dependency leads to a huge psychological and physical burden for the patient [17].

Due to insufficient patient-response-to treatments and various undesirable negative side effects, more and more scientific researchers in the medical area have begun to consider PPI step-down therapy in recent years. PPI step-down therapy means gradually reducing dosage and patients’ dependence on PPIs according to the outcome and symptoms changes of patients. Multiple strategies may be used to de-escalate (i.e., stop or reduce) PPIs, including abrupt PPI withdrawal, step down therapy, and H_2_ blocker (i.e., famotidine) substitution. However, one randomized controlled trial (RCT) study showed that about 68% patients experienced symptom recurrence after discontinuation of long-term PPI therapy [18], thus, meaning that stopping PPIs is not sufficient to control patients’ symptoms in a long-term period.

The use of complementary and alternative approaches has proven to have an effect on the treatment of NERD [19, 20]. In recent years, numerous studies have evaluated the clinical effectiveness of traditional Chinese Medicine (TCM) herbals for the treatment of NERD. A review article showed that in addition to their acid-suppressive properties, anti-inflammatory, and antioxidant activity of the herbal remedies, these herbal medicine seem to provide a safer and more effective treatment compared to their pharmaceutical drug counterparts. Herbal remedies used in human studies have led to the alleviation of heartburn and regurgitation related to NERD where PPI treatment has failed to show a promising effect. Moreover, herbal remedies appeared to have a longer-lasting therapeutic effect than conventional anti-secretory agents have [19]. A systematic review has reported that the total effective rate of TCM alone was superior to single PPIs or prokinetics for their clinical efficacy. TCM also has advantages in reducing recurrence rate and adverse events like constipation, faint headache experience, and nausea [21]. Although TCM has been examined for its ability to alleviate PPI-refractory symptoms, including the reduction of recurrence of these symptoms as well as improve the quality of life of patients, a high-level evidence-based support and thorough understanding of the underlying mechanisms of the action of TCM herbal remedies were lacking [22].

Tongjiang (TJ) methodology is used in TCM to treat functional gastrointestinal diseases. TJ literally means unobstructed and descent, and the main goals of TJ methodology in TCM are to regulate visceral dysfunction, adjust the imbalance between internal and external energies, restore the physiological functions of the viscera, and revitalize the mind, spirit, and body for a holistic well-being balance. Herbal granules based on TJ methodology can help regulate cell-to-cell tonic equilibrium and improve the overall Qi and food passing unimpeded through the entire gastrointestinal tract. Prior to this trail, we have performed an RCT study on the efficacy of TJ granule to improve the quality of the results and to provide estimates for further sample size calculation. The results showed that TJ granule could improve the symptoms which included heartburn, chest pain, acid or bitter tastes in mouth, and nausea. On the quality of life, it could also improve the domains of physical functioning and general health, and no adverse event was found [23].

For most common patients presented with NERD [17], we will use TCM granules based on the TJ methodology in cooperation with PPI step-down therapy to treat NERD. We designed this rigorous multicenter, double-blinded, RCT using TJ methodology granules with PPI step-down therapy for NERD patients with regard to NERD-related symptoms and symptom distress, to explore its therapeutic efficiency, measure recurrence rate, understand the mechanisms of the therapy, and estimate PPI withdrawal rate.

We aimed to independently examine the efficiency and safety of TJ methodology granules combined along with PPI step-down therapy for NERD patients with regard to NERD-related symptoms and symptom distress. The primary objective is to develop a new therapy for the treatment of NERD, to obtain high-level evidence-based medical evidence. The secondary objective is to evaluate the advantages of the new therapy, hoping to solve the problems of side effects and dependence caused by long-term use of PPIs. The last objective is to explore some of the mechanisms based on observations of the changes in patients’ gut microbiota.

## Method

### Study design

**Fig. 1:**
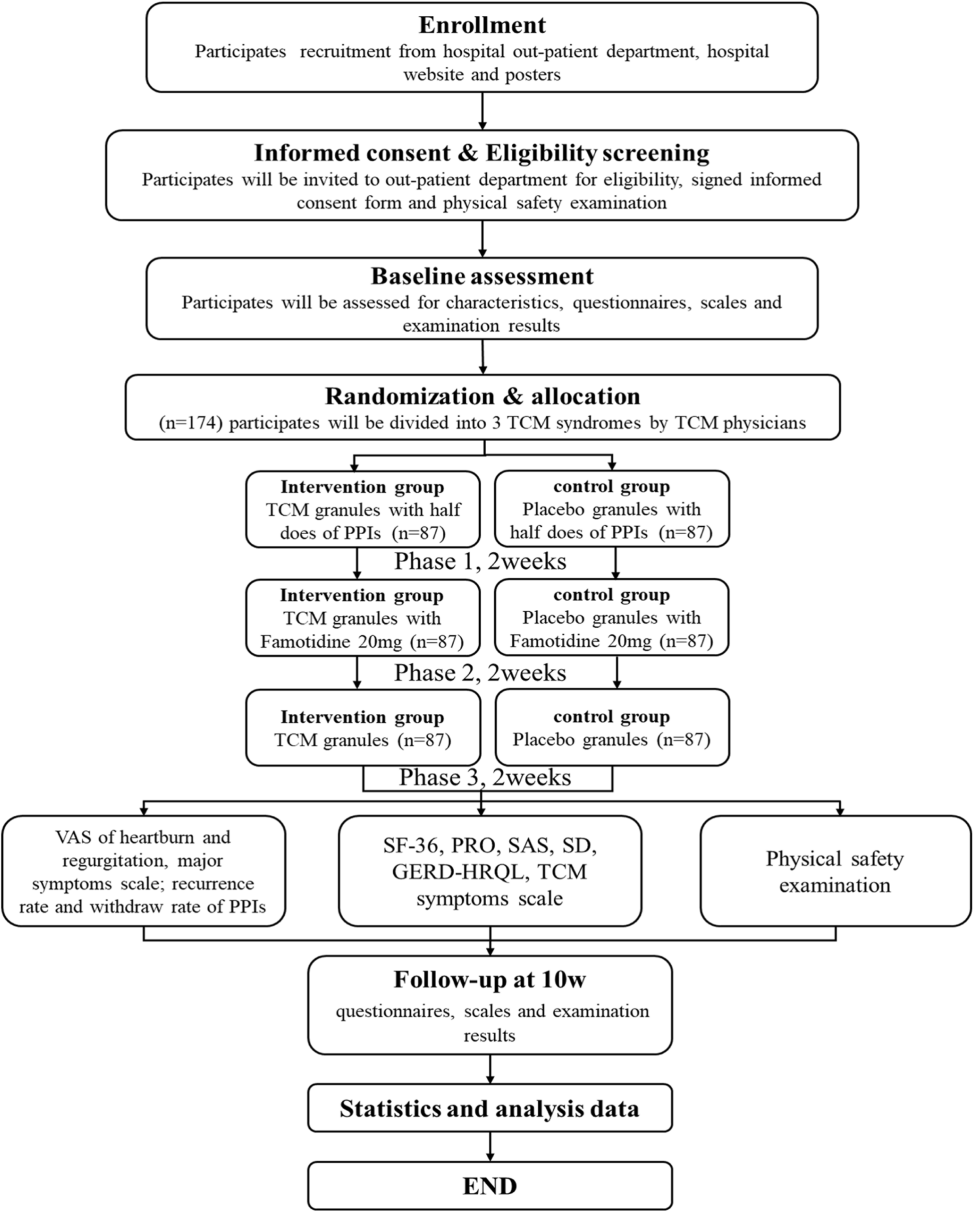
Flowchart of the proposed trial. VAS, visual analog scale; TCM, traditional Chinese medicine; SF-36, the MOS 36-item Short From Health Survey; PRO, patient-reported outcome; SAS, Self-Rating Anxiety Scale; SDS, Self-Rating Depression Scale; GERD-HRQL, the Gastroesophageal Reflux Disease-Health-Related Quality-Of-Life

This trial will adopt a multicenter, double-blinded, randomized controlled trial with two parallel groups, to explore the efficiency of the TJ methodology granules combined with PPI step-down for NERD. The protocol will be described in accordance with Recommendations for Interventional Trials 2013 guidelines [24]. The details of the study design are shown in Fig. 1.

### Study setting

This multicenter, double-blind, RCT will be conducted at six hospitals in China (Xiyuan Hospital of China Academy of Chinese Medical Sciences; Guangdong Provincial Hospital of Traditional Chinese Medicine; Wuhan No.1 Hospital; The First Affiliated Hospital of Tianjin University of Traditional Chinese Medicine; Traditional Chinese Medicine Hospital of Kunshan; Liuzhou Hospital of Traditional Chinese Medicine). These six hospitals are all qualified to conduct clinical studies and relevant experience. A total of 174 participants will be recruited from the hospital outpatient departments, hospital websites, and posters. Participants will be divided into three groups according to their different TCM syndromes, and those with the same syndrome will be divided into the intervention group and the control group at a 1:1 ratio.

### Eligibility criteria

All participating patients must first be judged by TCM physicians to determine whether they meet the following inclusion and exclusion criteria.

#### Inclusion criteria

1. The following are the diagnostic criteria of NERD according to the *China Consensus Opinion on Gastroesophageal Reflux Disease, 2014 edition*: (1) patients with typical heartburn and regurgitation that negatively affect the patient’s quality of life, mild symptoms for ≥ 2 days; (2) GERD Q scale score ≥ 8 points; and (3) NERD is diagnosed when there is no damage to the esophageal mucosa on gastroscopy within 1 month, esophageal 24-h pH, and impedance monitoring for pathological reflux within 1 year, and reflux esophagitis, Barrett’s esophagus, functional esophageal disease, and other upper gastrointestinal diseases are excluded
2. The diagnostic criteria of stagnation of liver and stomach heat syndrome or spleen deficiency and dampness-heat syndrome intermingled heat and cold syndrome in TCM. Major symptoms: (1) heartburn and (2) regurgitation
3. Treatment with PPIs for more than 8 weeks and whose curative effect is not satisfactory
4. Agreement to participate in the clinical trial and sign the informed consent form
5. Age from 18 to 65 years old

#### Exclusion criteria

1. Pregnant or breastfeeding females or those who are planning pregnancy
2. Other chronic GI diseases like RE or BE or primary diseases such as peptic ulcers, achalasia, pyloric obstructions, and esophageal cancer that can cause gastroesophageal reflux
3. Severe cardio-cerebrovascular system diseases, liver, kidney, hematopoietic system diseases, psychiatric diseases, and tumors
4. Anti-reflux surgery (fundoplication or endoscopic minimally invasive surgery) and other upper gastrointestinal surgery records
5. History of allergies or drug and food allergies appearing during the trials
6. Inability to describe self-symptoms or to cooperate with treatment
7. Abuse of alcohol or drugs, frequent changes in the working environment and tendency to dropout
8. Participation in other clinical trials within 4 weeks

#### Dropout criteria

1. Decision to exit the trial himself/herself
2. Loss to follow-up
3. Poor compliance
4. Diagnosis with other TCM syndromes or an order to withdraw by the TCM physicians

For the occurrence of patients presented with the above situation, we will consider making a suspension. In cases of dropout, the reason should be explained. If there are baseline pharmacodynamic data, the last main therapeutic effect index can be transferred to the final results for statistical analysis, and the CRF should be retained.

### Intervention

Each center’s treatment of participants will be provided by licensed TCM physicians. TCM physicians will distribute granules to participants tailored according to their symptoms under the guidance of the TJ methodology. To reduce intervention bias, TCM physicians have conducted relevant training. Our record physicians must study and be familiar with the scales and questionnaires in this trial and learn the skills to guide participants to complete them appropriately. The granule-distributing physicians arrange and distribute the granules accordingly. In addition to that, we have a chief physician responsible for overseeing the entire process.

#### Intervention group

Based on the curative effect of treating NERD in clinical practice, we selected three kinds of TCM granules based on TJ methodology. According to the different TCM syndromes, participants with stagnation of liver and stomach-heat syndrome will be treated with Tongjiang (TJ) granule, those with spleen deficiency and dampness-heat syndrome will be treated with Jianpiqinghua (JQ) granule, and those with intermingled heat and cold syndrome will be treated with Wenpiqingwei (WQ) granule. These herbal granules are available from the Xiyuan Hospital as an in-hospital preparation. TCM granules are concentrated herbal particles obtained by the mean of spray drying the hot water extract of a mixture of raw herbs. TCM granules are extracted with water to make an aqueous extract or decoction used for treatment. Each bag of granules (approximately 20 g) will be dissolved in 200 ml water and taken twice a day, 1 h after meal. Participants in all three groups cooperated with PPI step-down therapy. From week 1 to week 2 of treatment, the dose of PPIs will be half of the original dose, once a day, taken before bedtime. The PPIs will be replaced with famotidine 20 mg from week 3 to week 4, taken before bedtime. From week 5 to week 6, acid-suppressive drugs will be withdrawn, and only TCM granules will be administered. There will be 6 weeks of treatment and 4 weeks of follow-up.

#### Control group

Patients will be treated with the corresponding TJ methodology granules placebo. The placebo granules consist of cyclodextrin and 5% real TJ theory granules to imitate the color and smell of the real counterpart. Both herbal granules and placebo are manufactured by China Resources Sanjiu Medical and Pharmaceutical Co., Ltd. (Hefei, China; Batch number: 2004902) according to the standards of the Good Manufacturing Practice. The physical properties of the placebo, such as appearance, size, color, dose form, weight, taste, and smell, will be similar to those of the TCM granules. The PPI step-down therapy will be the same as that for the intervention group.

### Cointerventions

One week prior to the start of treatment, patients will be required to stop their intake of other drugs related to TCM therapies of NERD; however, acid-suppressive drugs intake will remain unchanged. During the study period, patients will be forbidden to add any drugs other than study therapies without permission. During the 1–4 weeks of the treatment period, if the participant’s symptoms increase after the reduction of PPIs according to the study protocol and cannot be tolerated, participant can temporarily use almagate suspension or almagate chewable tablets and record the combined medication. During the treatment period of 5–6 weeks, if the participant is unable to tolerate the symptoms after stopping the acid suppressive drugs, participant will be terminated from the trial and recorded and will be treated with their original treatment therapy.

### Timeline

The entire trial period will last 10 weeks (the treatment period is 6 weeks and the follow-up period will last 4 weeks). Participants will need to stop using drugs related to the treatment of NERD during pre-screening and screening, complete a physical safety examination, and sign an informed consent form. At baseline, participants will need to complete the first assessment and then randomly divided into an intervention group and a control group. During the treatment phase, TCM physicians will instruct the participants on how to take the therapy and then evaluate compliance and outcome indicators after 2, 4, and 6 weeks of treatment. The follow-up period will last 4 weeks after the end of treatment. Details are shown in Table 1.

**Table 1:** Timeline of the trial

Study period										
	Screening	Baseline	Treatment						Follow-up	
Outcomes	Week -1	Week 0	Week 1	Week 2	Week 3	Week 4	Week 5	Week 6	Week 8	Week 10
**Screening and enrolment**										
Informed consent form	○									
Eligibility for study	○									
Demographic and clinical details	○									
Physical safety examination	○									
Randomization	○									
**Intervention**										
TCM granules/placebo + PPI step-down		○		○		○				
**Assessment**										
VAS of heartburn and reflux		○	○	○	○	○	○	○	○	○
Major symptoms scale		○	○	○	○	○	○	○	○	○
Minor symptoms scale		○	○	○	○	○	○	○	○	○
TCM syndromes scale		○		○		○		○	○	○
SF-36		○		○		○		○	○	○
PRO for chronic gastrointestinal diseases		○		○		○		○	○	○
SAS		○		○		○		○	○	○
SDS		○		○		○		○	○	○
GERD-HRQL		○		○		○		○	○	○
Compliance			○	○	○	○	○	○		
Cointerventions			○	○	○	○	○	○		
Adverse events			○	○	○	○	○	○		

*VAS* visual analog scale, *TCM* traditional Chinese medicine, *SF-36* the MOS 36-item Short From Health Survey, *PRO* Patient-Reported Outcome, *SAS* Self-Rating Anxiety Scale, *SDS* Self-Rating Depression Scale, *GERD-HRQL* the Gastroesophageal Reflux Disease-Health-Related Quality-Of-Life

### Sample size estimation

According to the preliminary trial results from the team and to literature reports, the average effective rate of traditional Chinese medicine treatment for NERD is 90% [25]. The literature reports that the effective rate of PPIs in the treatment of NERD is between 50 and 90% [4, 26], so we used an average of 70%. The trial intends to conduct a superiority test of two independent sample proportions, using a margin of 0.02, an alpha risk of 0.05, and a beta risk of 0.20, assuming an attrition rate of approximately 20%. The estimated sample size is *N* = 174, including 87 cases in the intervention group and 87 cases in the placebo group.

### Randomization and blinding

In this trial, center stratification will be used; each center will use the block randomization method, and qualified participants will be divided into intervention and control groups in a 1:1 ratio for grouping. Sequence generation is as follows: a table of random sampling numbers will be completed by an independent statistician through the PROC PLAN procedure in the Strategic Applications Software (SAS) V.9.2, and the results of the random allocation will be published through the network central random allocation system. The statistician will place the groups to be assigned into a double-layered, opaque envelope in sequence and seal the envelopes, and the clinical research coordinator will number the envelopes sequentially and maintain them. Allocation concealment mechanism is as follows: the person who generates the random sequence and decides on the allocation sequence should not be involved in the inclusion of participates and should not be involved in subsequent trials, especially in the measurement of outcomes. Prepare a list of assignment sequences, with each column containing the subject’s serial number, random number, drug name, or group identification. It is important that the serial number of each included subject corresponds to the random number generated in this order and that the order is not changed. Determine the group based on the random numbers. The participants’ sequence number and corresponding grouping will comprise the first-level blind code and, the medicine code will comprise the second-level blind code; each patient’s medicine code will be randomly assigned. Implementation is as follows: the designer, pharmacy, or statistician will each be provided with a copy of the distribution schedule, each sealed in an opaque envelope and kept under lock and key. The placebo used in the control group is treated to look, smell, and even taste similar to the drugs in the treatment group, making it difficult to distinguish. Participants, drug distribution center staff, and trial personnel were unable to determine the type of drug based on its appearance. Each participant will receive the corresponding medication according to their random code. All operations will be completed according to the established SOP guidelines. All codes will be kept in the Good Clinical Practice (GCP) Centre of Xiyuan Hospital. The TCM practitioners and statisticians administering the treatment will be blinded to the grouping.

In the case of serious adverse events or complications in the clinical trials, blinding should be unsealed urgently, and cases of unblinding should be treated as dropout participates.

### Compliance

The patient’s compliance will be calculated as “actual dose/dose to be taken”; if the compliance is in the range of 0.7 to 1.2, it will be regarded as good compliance. Participants should return unused medicine and empty packaging at each visit, and TCM physicians will count the number of drugs and granules returned and keep a record of lost and unreturned medicines.

### Auditing

The clinical trial contract is drafted according to the contract template provided by the Good Clinical Practice institution, with the purpose of effectively protecting the safety of the subjects, and we shall be responsible for reporting the results of data safety monitoring to HRPPs on a regular basis. After the trial is completed, if there is a situation where the trial results directly affect the safety of the subjects, we have the responsibility to inform the investigator and the research institution, etc., of the results of the trial in a timely manner. The contract is initially reviewed and costed for the trial project by an institutional office independent of the drafter and signatory, and then reviewed. The clinical trial contract is signed under the premise of fairness, impartiality and openness.

### Ethics and dissemination

#### Patient consent

The trial is approved by the Ethical Review Committee of Xiyuan Hospital of China Academy of Chinese Medical Sciences. For eligible participants, a TCM physician will provide written informed consent forms. After the physician communicates the details of the study, participants willing to participate in the trial will sign an informed consent form. Participates and the TCM physician will each keep a copy of the form.

#### Confidentiality

All materials related to the trial will be safely stored in the restricted access location. All participant case reports and electronic charts will be kept confidential, and only authorized researchers will be able to access the paper version and electronic data set.

#### Protocol amendments

The trial protocol may be modified if we deem it necessary as new information becomes available during the trial or if we need to or management requests it. Once a modification is made to the trial protocol, re-approval from the Ethical Review Committee is required. If for ethical reasons (e.g., low efficacy, serious adverse events) or if the certainty of the trial becomes unacceptable, we will terminate the trial early.

### Outcomes and analysis

Participants in both the intervention group and control group will be encouraged to complete the treatment in 6 weeks, and follow-up will continue for 4 weeks. The following outcome indicators will be used to evaluate efficacy and safety. Demographic and clinical details of participants will be collected at the beginning of the trial. Physical safety examinations will be conducted at the beginning and end of the study, including (1) routine tests of blood, urine and stool, and fecal occult blood; (2) liver function tests (ALT, AST) and kidney function tests (Cr, BUN); and (3) electrocardiograms. Outcome indicators for efficacy evaluation will include the following: (1) a visual analog scale (VAS) for heartburn and regurgitation, (2) a major symptoms scale, (3) PPI withdrawal rate and symptoms recurrence rate, (4) a minor symptoms scale, (5) the MOS 36-item Short From Health Survey (SF-36), (6) the Patient-Reported Outcome (PRO) scale for chronic gastrointestinal diseases, (7) Self-Rating Anxiety Scale (SAS), (8) Self-Rating Depression Scale (SDS), (9) the Gastroesophageal Reflux Disease-Health-Related Quality-Of-Life (GERD-HRQL) scale, and (10) the TCM syndromes scale. Scales and questionnaires will be completed at the beginning and end of the trial and at the end of each phase and follow-up. Adverse events will be observed throughout the treatment.

### Primary outcomes

The primary outcomes are the VAS for heartburn and regurgitation and the major symptoms scale.

1. The VAS is a line marked with 10 scales, with 0 points to 10 points at both ends. Zero points indicates no symptoms, and 10 points represents the most severe and unbearable symptoms. 0–2 is classified as “basic no symptoms,” 3–5 is classified as “mild,” 6–8 is classified as “moderate,” and 8–10 is classified as “severe” [27–29]. Patients will be given a VAS for regurgitation and heartburn daily through a diary card, and the average score of the two symptoms each week will be evaluated according to the content of the diary card. Participants were defined as responders if for at least 50% of weeks during the 6 weeks of the trial the weekly VAS score was reduced from baseline by ≥ 50%. We will calculate the percentage of responders. At the same time, the change in VAS score will be compared between groups.
2. Major symptoms scale: For the main clinical symptoms of NERD, heartburn frequency, heartburn relief time, and regurgitation frequency, the scoring criteria are shown in Table 2.

**Table 2:** Major symptoms scale

	Score		
	Mild (0)	Moderate (1)	Severe (2)
Symptom			
Heartburn frequency	1 day a week	2–3 days a week	4–7 days a week
Heartburn relief time	Relieve within 1h	Relieve within 1-3h	> 3 h does not relieve even all day
Regurgitation frequency	1 day a week	2–3 days a week	4–7 days a week

### Secondary outcomes

Secondary outcomes are the PPI withdrawal rate and symptoms recurrence rate, scales about minor symptoms, health-related quality of life, mental health, and TCM syndromes.

1. PPI withdrawal rate and symptoms recurrence rate: During patient follow-up for the 4 weeks after the end of the treatment, according to the above scores, the number of recurrences and corresponding recurrence rates. The PPI withdrawal rate will calculated based on the number of people who no longer use PPIs at follow-up.
2. Minor symptoms scale: Scoring the frequency and degree of clinical minor symptoms. For the frequency, 0 points indicates no symptoms, 1 points indicates < 1 day per week, 2 points indicates 1 day per week, 3 points indicates 2–3 days per week, 4 points indicates 4–5 days per week, and 5 points indicates 6–7 days per week. For the degree of symptoms, 1 points means no symptoms; 2 points means symptoms are mild; 3 points means symptoms are moderate, need to take medicine occasionally; 4 points means symptoms are severe, need to take medicine for long time; and 5 points means symptoms are very serious, and affecting daily life, need to take medicine for long time. Details are shown in Table 3.
3. SF-36: As a concise health questionnaire, the SF-36 comprehensively evaluates the quality of life of the respondents from 8 dimensions including Physical Functioning (PF), Role-Physical (RP), Bodily Pain (BP), General Health (GH), Vitality (VT), Social Functioning (SF), Role-Emotiona (RE), and Mental Health (MH). It also contains another health dimension: Health Transition (HT), which is used to evaluate the overall change in health in the past year [30–33].
4. PRO: This scale is for patients with FGIDs. It contains a total of 35 items, divided into 6 dimensions (regurgitation, dyspepsia, general condition, social function, defecation, psychology) to assess the influencing factors of chronic gastrointestinal diseases. Each item is graded on a 5-point scale, with a maximum of 4 points and a minimum of 0 points. The higher the score, the more severe the symptoms [34, 35].
5. SAS: The SAS scale is a 20-item self-reported assessment device. Each question is scored on a Likert-type scale of 1 to 4 (based on the following replies: “a little of the time,” “some of the time,” “a good part of the time,” and “most of the time”). The total score is obtained by summing the assessment of the 20 items. The total score multiplied by 1.25 gives the standard score. A standard cutoff score of 50 is usually used to diagnose anxiety. The standard score ranges are 25–49 (normal range), 50–59 (mild anxiety), 60–69 (moderate anxiety), and 70 (severe anxiety) [36, 37].
6. SDS: The Self-Rating Depression Scale (SDS) developed by Zung is a norm-referenced measure, used to screen adults for the potential presence of depressive disorders. The scale produces raw scores between 20 and 80; Zung recommended converting these to Index Scores (which ranged between 25 and 100) by the simple process of multiplying by 1.25. Zung’s recommended cut-off for identifying adults with depressive disorder was index scores of 50 and over [38]. We used in this study an index score of 53 (raw score 42); this was more appropriate for use with Chinese populations [39].
7. GERD–HRQL: A total of 16 items are used to evaluate the symptoms of heartburn, regurgitation, and the effects of current medication on life. Each item is scored on a 6-point scale, from 0 to 5 points. A score of 0 indicates no symptoms, and a score of 5 indicates that symptoms are incapacitating when performing daily activities [40, 41].
8. TCM syndromes scale: a 15-item scale based on *the Clinical Guideline of New Drugs for Traditional Chinese Medicine*. This scale evaluates the changes in TCM symptoms of the digestive tract and the whole body of the participants, including flatulence, stomachache, poor appetite, heartburn, belching, acid reflux, pharyngeal paraesthesia, thirst, distention in the lateral lower abdomen, fatigue, shortness of breath, unwillingness to speak, somatosensory heaviness, fear of cold, and loose stool. Each item is scored, with 1 being asymptomatic and 4 being severe. The higher the overall score, the more severe the overall symptoms [42].

**Table 3:** Minor symptoms scale

Symptoms	Frequency score	Degree score
Non-cardiogenic chest pain		
Epigastric pain		
Epigastric discomfort		
Belch		
Cough		
Asthma		
Pharyngeal paraesthesia		
Total		

### Exploring outcomes

At baseline and after 6 weeks of treatment, fecal samples will be collected and stored at – 80 °C for unified testing. The experimenter will extract total DNA from the fecal samples and perform 16S rRNA sequencing. Reads will be processed to generate an operational taxonomic unit (OTU) table at 97% granularity. Through OTU analysis, the detected α-diversity and β-diversity of the fecal microbiome will be detected, and then the LEfSe analysis will be used to compare and analyze the data to identify species with significant differences in abundance between groups.

### Data management and monitoring

We will use both written and electronic CRF. At each time point, participants will complete the CRF under the guidance of a TCM physician. The completed CRF will be entered into the Drug & Clinical Trial Data Management Platform (developed by the GCP center: http://www.xyedc.com/). The CRF data will be entered in duplicate by two trained TCM physicians. Electronic data will be permanently saved. After the study, the statisticians will be able to download the electronic data for analysis through the platform.

The Data Monitoring Committee (DMC) reviews some of the data in the form of interim analyses to make preliminary judgments about the efficacy and safety of the study drug. The DMC is responsible for ensuring the safety and benefit of the subjects, the integrity and credibility of the trial, and the timely and accurate feedback of study results to the relevant areas of clinical research. The monitoring plan foresees the conduction of a total of four monitoring visits from the point of selection of the first study patients to the end of the regular monitoring visits. After charting all adverse events reported during the study period, the incidence of adverse events will be determined.

### Statistical analysis

This trial will use intention-to-treat (ITT) analysis as the main method to evaluate the outcome results. To deal with missing outcome variables that remain to be recorded, the last record data will be carried over to the end or a multiple imputation method will be used.

Statistical analysis will be conducted using the SPSS 21.0 software. The significance level is established at 0.05, and the limits of the confidence interval are 95%. Descriptive analysis and categorical variables will be described in frequency tables and as percentages or composition ratios; continuous variables will be described in terms of means and standard deviations (SDs), or medians, lower quartiles (P25), upper quartiles (P75), minimum values, and maximum values. Comparative analyses between two groups will be performed for categorical variables using the chi-squared test, Fisher’s exact probability method, the Wilcoxon rank-sum test, or the CMH test. Continuous variables that conform to a normal distribution will be compares using a *t*-test (for homogeneity of variance between groups, with 0.05 as the test level; if the variance is heterogeneous, the Welch-Satterthwaite *t*-test will be used), while those that conform to a non-normal distribution will be compared using the Wilcoxon rank-sum test or the Wilcoxon signed-rank test. Hypothesis testing will use the two-sided test uniformly, and *P* ≤ 0.05 will be considered statistically significant.

## Discussion

To date, there are increasing numbers of patients negatively affected by NERD symptoms, which also impose a heavy economic and life burden to patients [43]. This trial will use TCM granules combined with PPI step-down therapy as the intervention group and granule placebo combined with PPI step-down as the control group to study the efficiency and safety of the TJ methodology series granules combined with PPI step-down for treating NERD patients. This trial aimed to explore the possibilities whether the dosage of PPIs can be significantly reduced after treatment with TCM granules and observe the recurrence of the disease after treatment.

Based on multiple clinical researches and scientific systematic reviews in recent years, when compared to the treatment of western medicine alone, TCM has superior advantages in the treatment of functional gastrointestinal diseases, which is proven to improve treatment effectiveness rates and reduce recurrence rates and side effects; however, there were just small amount of them analyzing and looked thoroughly into the recurrence rate [21, 22]. The quality of about 90% TCM RCTs was considered poor, lacking of descriptions of blinding and randomization protocols in studies, neglecting stringent diagnostic guidelines and therapeutic criteria evidence [44]. This has led to its received skepticism by modern medicine society due to the lack of high quality trials. In previous reviews, there were basically no studies related to PPI step-down was retrieved and recorded; therefore, in this trial, we will conduct a rigorous randomized, double-blind clinical trials to clarify the efficacy of TCM granules derived from TJ methodology combined with PPI step-down and will perform quality of life, mental health, recurrence, and PPI withdrawal rate of the therapy.

We regard this trial with upmost importance due to the facts that 20–30% of NERD patients do not respond to PPI treatment [45] and that the long-term use of PPIs produces dependence and negative side effects [46, 47]. With the cooperation of TCM granules and PPI step-down therapy, it can synergistically adjust the holistic internal and external energy of NERD patients. On the premise of relieving symptoms, gradually reduce the dosage of acid suppressive drugs every 2 weeks to withdraw. It can provide a solution to the problem of NERD patients not responding to PPIs and reduce the side effects caused by long-term use of PPIs. Experiments had proved of the effect of granules based on TJ methodology on increasing the level of plasma motilin, reducing the level of gastric acid, and promoting the gastric emptying. The acute toxicity experiment showed that there was no toxic reaction in mice [23].

This trial will use VAS for heartburn and regurgitation, major and minor symptoms scales, SF-36, PRO, SAS, SDS, GERD–HRQL, TCM syndromes scales, and symptom recurrence rate to measure physiological and psychological outcomes to comprehensively evaluate the changes imposed by this therapy for the participants before, during, and after treatment and the PPI withdrawal rate, in order to provide high-level medical evidence for the TCM treatment of NERD and to form a new therapy regimen for NERD. For patients with long-term use of PPIs that are unable to withdraw, the advantages of the new therapy and the mechanisms related to gut microbiota will be evaluated. This trial is expected to solve the side effects and dependence caused by long-term use of PPIs and compare the gut microbiota diversity of healthy volunteers and NERD patients before and after treatment to prepare for the next step of exploring the underlying mechanism of the disease. The results of this trial will help to improve the quality of evidence of TCM studies and the clinical efficacy of NERD treatments, reduce dependence on PPIs, and improve the quality of life of patients, ideally yielding good social benefits.

## Trial status

Protocol version number and date: XYYY-KY-NERD-03, 22 February, 2020

The date recruitment began: 15 June, 2020

The approximate date when recruitment will be completed: 31 December 2022

## Data Availability

Not applicable. This is a protocol; no data are generated.

## Abbreviations

AET: Acid exposure time
BE: Barrett’s esophagus
BP: Bodily pain
EE: Erosive esophagitis
GERD: Gastroesophageal reflux disease
GERD-HRQL: The Gastroesophageal Reflux Disease-Health-Related Quality-Of-Life
GH: General health
HT: Health Transition
MH: Mental health
NERD: Non-erosive reflux disease
PF: Physical functioning
PPIs: Proton pump inhibitors
PRO: Patient-reported outcome
RCT: Randomized controlled trial
RE: Role-Emotiona
RP: Role-Physical
SAS: Self-Rating Anxiety Scale
SDS: Self-Rating Depression Scale
SF: Social Functioning
SF-36: The MOS 36-item Short From Health Survey
TCM: Traditional Chinese medicine
TJ: Tongjiang
VAS: Visual analog scale
VT: Vitality
